# Resistance to Chemical Attack of Hybrid Fly Ash-Based Alkali-Activated Concretes

**DOI:** 10.3390/molecules25153389

**Published:** 2020-07-27

**Authors:** William G. Valencia-Saavedra, Ruby Mejía de Gutiérrez

**Affiliations:** Composites Materials Group (CENM), Universidad de Valle, Calle, Cali 13 #100-00, Colombia; william.gustavo.valencia@correounivalle.edu.co

**Keywords:** fly ash, hybrid concrete, alkali-activated cements, durability, sulphates, carbonation, chlorides

## Abstract

The environmental impacts related to Portland cement production in terms of energy consumption, the massive use of natural resources and CO_2_ emissions have led to the search for alternative cementitious materials. Among these materials, alkali-activated cements based on fly ash (FA) have been considered for concrete production with greater sustainability. In the present article, the chemical durability properties (resistance to sulphates, chloride permeability, and resistance to carbonation) of a hybrid alkali-activated concrete based on fly ash–ordinary Portland cement (FA/OPC) with proportions of 80%/20% were evaluated. It is noted that the FA was a low-quality pozzolan with a high unburned carbon content (20.67%). The results indicated that FA/OPC concrete had good durability with respect to the OPC concrete, with 95% less expansion in the presence of sodium sulphate and a 2% strength loss at 1100 days, compared with the 56% strength loss of the OPC concrete. In addition, FA/OPC showed lower chloride permeability. On the contrary, the FA/OPC was more susceptible to carbonation. However, the residual compressive strength was 23 MPa at 360 days of CO_2_ exposure. Based on the results, FA/OPC, using this type of FA, can be used as a replacement for OPC in the presence of these aggressive agents in the service environment.

## 1. Introduction

The durability of concrete structures exposed to different aggressive agents is a subject of great importance for safe and efficient construction with concrete [1]. The main factors that reduce concrete durability are chemical attacks, such as carbonation, chloride attack, and sulphate attack, phenomena that can lead to the corrosion of structural steel [2]. The rate and mechanism of the different attacks depend on factors such as the type of cement, design and curing of the concrete mixture, the permeability of the material, the concentration of the attacking medium, exposure temperature, and the nature of the products formed [1,3]. Different studies have demonstrated that the durability problems in Portland cement concretes are associated with the decalcification of C–S–H and the formation of new products [4,5]. The addition of Supplementary Materials to Portland cement systems, such as pozzolans and blast furnace slags, reduces the proportion of portlandite present in the mixture. These materials contribute to the refining of the pore structure, resulting in lower concrete permeability and consequently higher resistance to attack from certain aggressive agents [6,7]. The crystallization of salts in very fine pores can also cause stress phenomena and cracks, negatively impacting the mechanical properties of the structure [5]; in addition, the reduction of portlandite can accelerate carbonation phenomena.

Alkali-activated concretes are a new class of cementitious materials obtained from the alkaline activation of materials with high silica and alumina contents that can contain calcium, such as fly ash (FA), metakaoline (MK), and granulated blast furnace slag (GBFS) [8,9,10,11]. These materials have shown excellent mechanical properties, even at early ages. However, due to the variability in the type and sources of the precursors, some of which come from industrial by-products and/or wastes, it is necessary to study the durability of these materials in different environments in depth. Studies regarding the durability of these materials are scarce, and most of the relevant studies have been performed on pastes and mortars, many at variable concentrations and for relatively short exposure periods [12,13].

Škvára, Jílek, and Kopeckỳ [14] investigated the sulphate resistance of pastes and mortars up to an age of 720 days and reported the resistance increments throughout the tests, which indicated no dimensional changes nor the formation of expansion phases. These researchers presumed that sodium sulphate acted as an activator in the mixture. Puertas et al. [15], studied the behavior of slag mortars and FA/GBFS 50/50 mixtures activated with sodium silicate and sodium hydroxide, and in both cases, the activated mortars were highly resistant to sulphate attacks; however, the samples activated with NaOH were more susceptible to degradation due to the presence of expansive phases such as gypsum and ettringite. Ismail et al. [16], studied FA/GBFS 50/50 activated pastes exposed to sodium and magnesium sulphate at 5% for a period of three months and reported that magnesium sulphate was more aggressive due to the higher decalcification of the calcium silicate hydrate gel and the formation of gypsum, which coincides with results that have been reported for Portland cement concretes [5]. In general, FA-based systems with GBFS additions activated with a mixture of sodium hydroxide and silicate, have showed good resistance to sulphate attack (sodium and magnesium).

In contrast, the susceptibility to carbonation has been identified as a potential disadvantage of alkali-activated cements compared with Portland cement, given that in different studies, significant structural changes occur in these materials in the presence of CO_2_, which could compromise the long-term performance of these materials [17,18,19,20]. Most of the studies have been performed under accelerated conditions using a chemically controlled mechanism, demonstrating that the effects depend on factors such as the specific microstructural characteristics, types of precursors and nature of the activator [21]. The test conditions, such as the relative humidity and CO_2_ concentration, also significantly impact the rate and degree of carbonation in these materials [17]. Despite the elevated susceptibility to carbonation shown by different alkali-activated systems under laboratory conditions, no correlation was observed with the results obtained by exposing these materials to atmospheric conditions (normal, 0.035% CO_2_, or reduced levels of CO_2_) over many years [22]. Thus, using the accelerated test method for evaluating the carbonation of alkali-activated materials can underestimate the real performance of these materials during their service life.

Studies regarding the performance of these materials in the presence of chlorides are scarce, and most of the studies have been performed on pastes and mortars. Rajamane et al. [23], studied the chloride ion permeability of alkali-activated systems based on GBFS and mixes GBFS+FA (25 and 50% FA) under ASTM standard C1202, and determined that these systems had low chloride ion permeability (722 to 1222 coulombs). Ganesan et al. [24], using the same technique, evaluated FA-based alkali-activated concrete (100%FA) exposed to chlorides and also reported low chloride ion permeability (1321 coulombs). Additionally, the authors using ASTM C1556 reported a chloride diffusion coefficient of 1.24·10^−11^ m^2^/s [24]. In contrast, a study by Olivia and Nikraz [25] reported that the chloride permeability of alkali-activated FA concrete is higher than that of ordinary Portland cement (OPC) concrete. Shayan et al. [26] characterized the properties of a GBFS-based alkali-activated system after being in service for five years in the retaining walls of a bridge structure over the Yarra River in Melbourne, Australia. The rapid chloride permeability test ASTM C1202 showed a very low chloride permeability. Ma et al. [27] and Tennakoon et al. [28] have obtained a chloride diffusion coefficient of alkali-activated systems lower than that of OPC concrete.

In recent years, other types of alkali-activated concretes containing percentages of up to 30% OPC have been considered, which are produced with a highly blended cement after being alkali-activated. These materials have been named “Alkali-activated Portland Blended cements, or Hybrid cements” [29]. The mechanical strength development of this type of hybrid material has been studied [30], however, to date no durability studies have been reported. Considering that these hybrid cements with a low Portland cement content and high proportion of aluminosilicates represent a more viable option in the immediate future, it is important to investigate their performance in aggressive environments.

Based on the above, the objective of this paper was to evaluate the behavior of an alkali-activated Portland fly ash concrete (FA/OPC; 80%/20%) under exposure to sulphates (Na_2_SO_4_ and MgSO_4_), CO_2_ (in an accelerated chamber under controlled conditions), and chlorides. The results are compared with those of a concrete produced with conventional cement (100% OPC) exposed to the same aggressive environments. It is noted that, most of the FA generated in Colombia, particularly that from industrial boilers, has showed a high carbon content due to a regular control of the combustion process, which limits its use. Therefore, this type of FA is considered a waste material and causes the contamination of the soil and atmosphere. The use of this material as a precursor of hybrid binders will allow to produce lower carbon footprint materials and increase the added value to the residue. The knowledge of its mechanical properties and durability will make its use in the civil construction sector more feasible.

## 2. Results and Analysis

### 2.1. Exposure to Sulphates

#### 2.1.1. Expansion Tests and Visual Inspection

**Figure 1:**
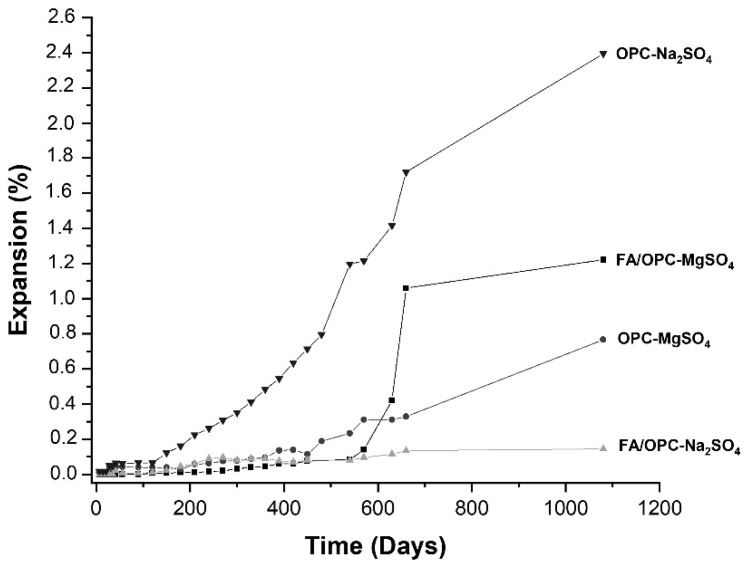
Expansion vs. time for the concrete exposed to Na_2_SO_4_ and MgSO_4_.

The changes in the length of the concrete bars (FA/OPC and OPC) after exposure to Na_2_SO_4_ and MgSO_4_, evaluated in accordance with the ASTM standard C1012 are included in Figure 1. The OPC samples were more affected than the FA/OPC samples in the Na_2_SO_4_ exposure medium, and the FA/OPC samples exposed to MgSO_4_ expanded more than the samples immersed in Na_2_SO_4_ at 450 days of exposure. At 28 days of exposure to the Na_2_SO_4_ solution, the OPC sample expanded by 0.050%, remaining approximately stable up to 120 days, unlike the FA/OPC samples, which expanded by only 0.011% at the same age, a value that is approximately five times lower than that of the OPC sample. From this age of exposure, the expansion of the OPC samples significantly increased, reaching an expansion six times higher than that of the FA/OPC (0.090%) at 360 days. At the final age of the test (1100 d), the expansion of the OPC samples again significantly increased (2.390%), while the expansion of the FA/OPC samples only slightly increased to 0.140%, which in general terms is 95% lower than that of the OPC concrete.

The FA/OPC samples immersed in the MgSO_4_ solution did not change size during the first 120 days of exposure, unlike the conventional OPC concretes, which expanded by 0.018%. At six months of exposure, the OPC concretes had expanded three times more than the FA/OPC concrete. From 630 days of exposure, the hybrid FA/OPC alkali-activated concretes expanded more than the OPC concrete, up to 30%. At the end of the test (1100 d), the expansion of the FA/OPC concretes was 1.60 times higher than that of the OPC in MgSO_4_.

The OPC-based concretes deteriorated more than the alkali-activated FA/OPC concretes from exposure to sodium sulphate. This is due to the reaction of the sulphate with calcium hydroxide and calcium monosulfoaluminates to form gypsum and ettringite, which generate the expansion, cracking, and detachment of the surface layers in the concretes, and a finally loss of strength [31,32]. Sata et al. [33], attributed the better performance of alkali-activated mortars to the lower susceptibility to sodium sulphate of the geopolymerization products, compared with the hydration products of the Portland cement. Bakharev [34] attributes this effect to the cross-linked polymer structure of aluminosilicate gel. Regarding the samples immersed in MgSO_4_, the alkali-activated concretes expanded more, which also coincides with the results of Bakharev [34] and Ismail et al. [16], who attributed this behavior to the decalcification of the C–A–S–H gel, forming gypsum.

**Figure 2:**
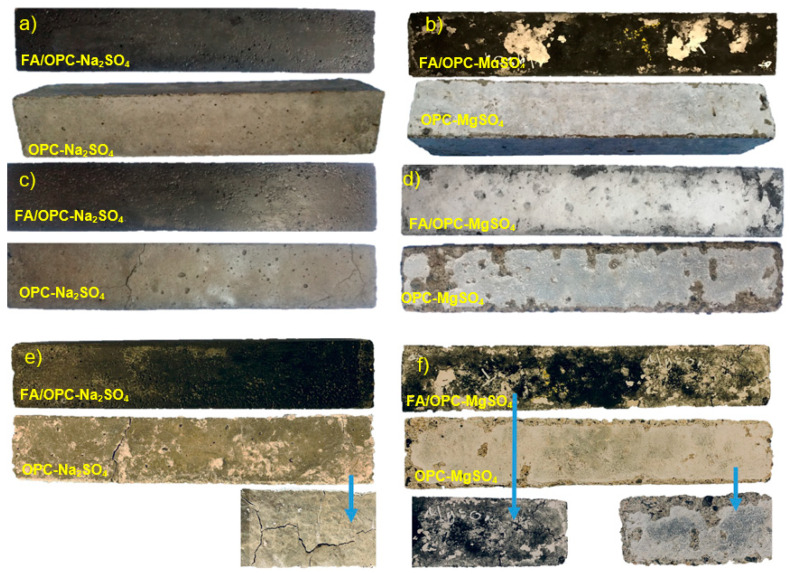
Concrete exposed: (**a**) Na_2_SO_4_ for 210 days; (**b**) MgSO_4_ for 210 days; (**c**) Na_2_SO_4_ for 440 days; (**d**) MgSO_4_ for 440 days; (**e**) Na_2_SO_4_ for 1100 days; and **f**. MgSO_4_ for 1100 days.

Figure 2 presents photographs of the concretes immersed in Na_2_SO_4_ and MgSO_4_ at exposure ages of 210, 440, and 1100 days; more damage was observed in the OPC specimens than the FA/OPC samples, and higher degrees of damage were seen in the samples exposed to the MgSO_4_ solution.

From an age of 210 days, the crumbling of the edges of the bars and attack on the surfaces were evident for the OPC specimens in the sodium sulphate solution, as shown in Figure 2a. Conversely, the FA/OPC specimens did not physically deteriorate, and only the formation of salts was observed on the surfaces with no effects on the integrity of the samples. At 440 days, as shown in Figure 2c, the edges of the OPC bars exhibited cracks, unlike the FA/OPC samples, whose surfaces did not change. In the magnesium sulphate solution, at the same age of exposure, the physical deterioration of the OPC bars significantly increased, and delamination was observed at the edges, as shown in Figure 2d. Furthermore, for the FA/OPC specimens, larger amounts of salt were deposited on the surface, and small surface cracks were observed (Figure 2b,d). These differences in behavior as a function of the type of sulphate solution used have been observed by other researchers in the pastes of FA/GBFS 50:50 binary mixtures [35] and fly ash-based geopolymer mortars [34].

Figure 2e shows the FA/OPC and OPC samples at 1100 days of exposure to sodium sulphate; the FA/OPC did not show signs of damage on the surface nor cracks, in contrast with the OPC concrete samples. This coincided with the low expansion values of the FA/OPC samples at this age of exposure (0.14%) compared with the 2.40% expansion of the OPC samples. Regarding the samples immersed in magnesium sulphate, the FA/OPC concretes deteriorated more than the OPC samples; these samples presented more cracks on the surface and crumbling of the edges (Figure 2f). This behavior was due to the formation of crystalline phases of gypsum, as observed by X-ray diffraction testing. The maximum expansion limit specified by ASTM C1012 and ACI C201 for Class 3 exposure (equivalent at severe exposition, ≥10.0000 ppm SO_4_^2−^) at 18 months was 0.10%; FA/OPC complies to this value under Na_2_SO_4_ (0.080%) and MgSO_4_ (0.084%) exposure. This performance allows its classification, according the standards mentioned above, as sulphates-resistant concrete.

#### 2.1.2. Effect on Compressive Strength

**Figure 3:**
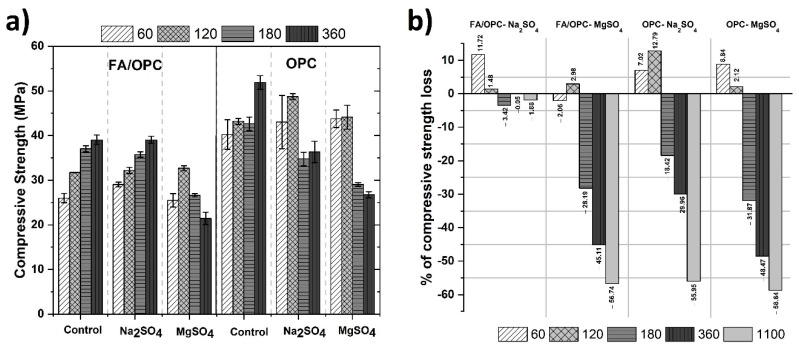
(**a**) Compressive strength and (**b**) loss of compressive strength of the concretes exposed to MgSO_4_ and Na_2_SO_4_.

Figure 3a, shows the development of the compressive strength of the concrete after exposure to the magnesium sulphate and sodium sulphate (MgSO_4_ and Na_2_SO_4_) solutions at ages of exposure up to 1100 days. The results obtained for the samples (FA/OPC and OPC) that were not exposed to sulphates (control samples) were also included in the same Figure. The results of each test correspond to a minimum of three specimens per age, and the deviations of the results range between 0.4 and 6%. In general terms, between 60 and 120 days of exposure to sodium and magnesium sulphates, the FA/OPC concretes increased in mechanical strength compared with the control samples by orders ranging between 2 and 12%. These results agree with those reported in the expansion tests, where relatively low values were observed at an age of 120 days (a maximum of 0.060% for the OPC samples and 0.005% for the FA/OPC samples). In Portland cement concretes, the initial increase in strength is supposedly due to the formation of new phases that act by filling the pores and densifying the material; however, since these phases expand, once the strength exceeds the tensile strength of the concrete, cracking occurs, and the strength drastically decreases [4].

For the alkali-activated materials, Baščarević et al., [3] claimed that sodium sulphate can act as an activator in the material, and consequently, the mechanical strength can initially increase and decrease over the long term, although by relatively low orders; the authors reported values of 12% at 360 days under exposure to sodium sulphate. In the current study, at the age of 180 days of exposure, under exposure to Na_2_SO_4_ and MgSO_4_, the mechanical strengths of the FA/OPC and OPC concretes decreased, as shown in Figure 3b; this reduction was higher in the presence of magnesium sulphate, and higher for the OPC samples. The compressive strength loss of the FA/OPC was 3.42 and 28.19% when exposed to Na_2_SO_4_ and MgSO_4,_ respectively. This behavior in the alkali-activated systems coincides with that reported in other studies and can be considered of minimal significance [16]. Regarding the OPC concrete, this behavior could be related to the formation of crystalline phases that cause expansion such as ettringite, which causes the cracking of the structure and an increase in the porosity [36]. At 360 days of exposure, the strength of the OPC in Na_2_SO_4_ was reduced by 30%; conversely, the strength of the FA/OPC samples did not significantly vary compared with the reference samples. In MgSO_4_, the strength of the FA/OPC concrete decreased by 45%, and that for the OPC concrete decreased by 48%. At the final test ages (1100 d), the strength of the FA/OPC samples exposed to sodium sulphate decreased by only 2%, unlike the OPC samples, which presented losses of 56%. For the samples immersed in magnesium sulphate, the strength decreased up to 58%; however, the behavior of the two types of concretes was similar. The mechanical performance of the concretes exposed to sulphates coincides to a high degree with what was observed in the expansion tests.

#### 2.1.3. Mineralogical Characterization of the Specimens by XRD and SEM

**Figure 4:**
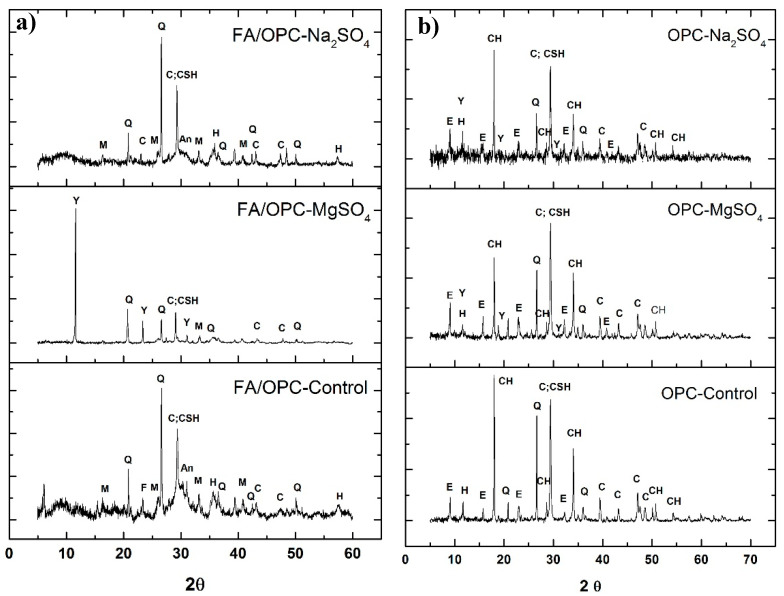
X-ray diffractograms for the pastes after 180 days of exposure. (**a**) FA/OPC and (**b**) OPC.

Figure 4 presents the X-ray spectra of the FA/OPC and OPC pastes after exposure to Na_2_SO_4_ and MgSO_4_ for a period of 180 days. The spectra of the FA/OPC-Control and OPC-Control are included for comparison purposes, which correspond to the reference pastes.

Crystalline phases that correspond to the raw materials, such as C: calcite, Q: quartz, M: mullite, and H: hematite, are observed in the FA/OPC-Control spectrum, in addition to the C–(A)–S–H main phase, which is located at approximately 30° 2θ, overlapping the calcite peak [37]. The FA/OPC samples immersed in MgSO_4_ present a new high-intensity phase (Y: gypsum) that can arise from the decalcification or decomposition of the C–(A)–S–H phase, which is corroborated by the lower intensity of the peak corresponding to the C–(A)–S–H phase [2,16,38]. The high intensity of the gypsum peak at 11.63° 2θ may suppress the visibility of other lower-intensity peaks of the different phases present. In the FA/OPC pastes exposed to Na_2_SO_4_, no new crystalline phases appear compared with the FA/OPC-Control, which agrees with the results obtained in the previous tests of expansion percentage and loss of mechanical properties. This result coincides with those reported by other researchers [3,16].

In the OPC-Control samples, hydration products are observed, CH: portlandite and E: ettringite, as well as the crystalline phases present in the original material (OPC) due to the addition of limestone (Q: quartz, C: calcite and H: hydrocalcite). The peak at 29.4° 2θ corresponds to the calcite present in the cement and overlaps with the peak attributed to low-crystallinity calcium silicate hydrate (CSH) [39]. Ettringite and gypsum peaks are present for the samples exposed to both media, with more intense peaks for the samples immersed in the MgSO_4_ solution. This result coincides with the decrease in the intensities of the peaks attributed to calcite, the CSH phase, and CH, which is also observed for the FA/OPC exposed to MgSO_4_ and is an indication of CSH decalcification. For the OPC concretes exposed to sodium sulphate, the formation of gypsum is reportedly associated with the formation of sodium hydroxide, which maintains a highly alkaline pH, contributing to the higher stability of the CSH. Conversely, in the presence of magnesium sulphate, magnesium hydroxide (brucite) is formed, which is an insoluble and poorly alkaline phase, so the stability of the CSH decreases, which is the main reason why MgSO_4_ is more aggressive. Alexander et al. [4], and Sata et al. [33], claimed that at a pH of 12–12.5, only ettringite forms, whereas at a pH between 8 and 11.5, gypsum forms, and decalcification is preferred.

**Figure 5:**
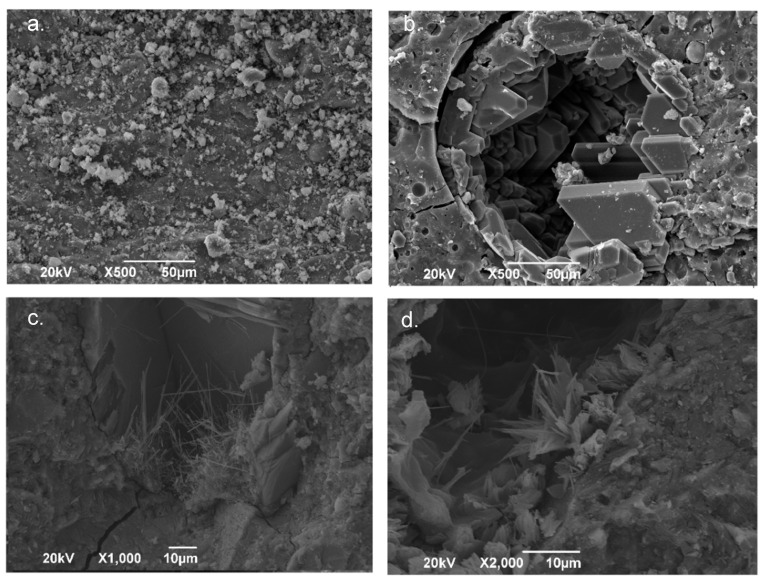
SEM micrographs of the pastes exposed for 180 days: (**a**) FA/OPC Na_2_SO_4_; (**b**) FA/OPC MgSO_4_; (**c**) OPC Na_2_SO_4_; and (**d**) OPC MgSO_4_.

Figure 5 presents SEM micrographs of the FA/OPC and OPC pastes exposed to the MgSO_4_ and Na_2_SO_4_ solutions for 180 days.

In the FA/OPC sample exposed to MgSO_4_, gypsum crystals were observed embedded in the alkali-activated matrix, as shown in Figure 5a. At a high magnification (10,000×), needle-type crystals were observed, due to the reduced proportion of ettringite. For the FA/OPC concretes immersed in the Na_2_SO_4_ solution, no new phases formed, and no specific changes were observed in the morphology, as shown in Figure 5b. Similar micrographs have been reported in other studies [16]. For the OPC pastes immersed in MgSO_4_ and Na_2_SO_4_, the SEM micrographs, as shown in Figure 5c,d, confirmed the presence of ettringite, which was observed in the X-ray diffraction analysis. These results agree with those presented by [33]. No evidence of gypsum was found in the OPC specimens exposed to MgSO_4_, though gypsum was indicated by the XRD analysis.

### 2.2. Susceptibility to Carbonation

#### 2.2.1. Carbonation Depth

**Figure 6:**
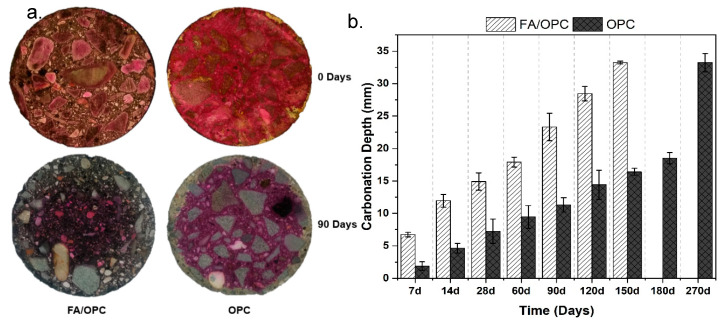
(**a**) Photographs of the concrete specimens at 90 days of exposure to CO_2_; and (**b**) Carbonation depth.

Figure 6a presents the samples that were cured for a period of 28 days and then exposed to CO_2_ 1% (HR = 65%, T = 25 °C) for a period of 90 days; a phenolphthalein indicator was applied to reveal the carbonated depth. In general, the OPC samples presented shallower carbonation depths. To more precisely observe the carbonated zones, measurements of these zones were taken in different directions, and an average carbonation depth was obtained at the ages of 7, 14, 28, 60, 90,120, 150, 180 and 270 days of exposure; the results are presented in Figure 6b. In general, the carbonation depth increased in all the samples evaluated as the age of exposure increased. This increment was more notable for the hybrid FA/OPC alkali-activated samples.

The carbonation coefficient was determined using the Equation (1), where *K_c_* is the carbonation coefficient, *X_c_* is the carbonation depth (mm) and t is the time of exposure (days) [40]:

(1) $$K_{C}=\frac{X_{C}}{\sqrt{t}}$$

**Figure 7:**
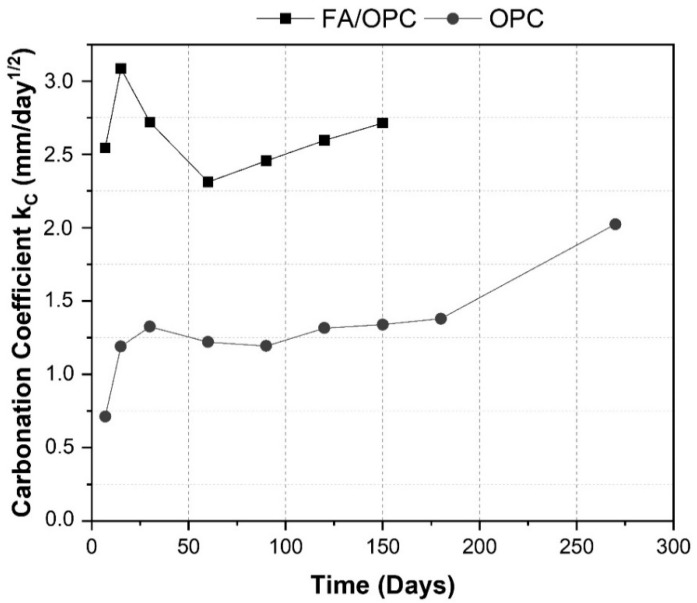
Concrete carbonation coefficient.

Comparing the carbonation depth with the carbonation coefficient (*K_c_*) in Figure 7, throughout the exposure period, the OPC concretes performed better than the FA/OPC samples. The carbonation coefficient of the FA/OPC concretes after 7 days of exposure was approximately 2.5 mm/day^1/2^, while that of the OPC samples was 0.71 mm/day^1/2^. At longer exposure times (150 days), the carbonation depth of the FA/OPC concretes (87%) was greater than that of the OPC samples, with carbonation depths up to 43%. This behavior coincides with that of the carbonation coefficient, where the FA/OPC samples presented a coefficient of 2.7 mm/day^1/2^ at 150 days of exposure, while the OPC concretes presented values of 1.3 mm/day^1/2^.

It is noted that the FA/OPC samples presented 100% carbonation at 180 days. The same behavior was observed by Behfarnia and Rostami [41], Bernal et al. [42], and Puertas et al. [20], for alkali-activated mortars and concretes based on blast furnace slag (GBFS) with respect to OPC concretes. These researchers attributed this behavior to certain chemical changes occurring in the matrix, such as the formation of calcium carbonate crystals, a decrease in the pH level of the pore solution, and the decomposition of the C–A–S–H gel formed. On the other hand, the OPC concretes or mortars have a higher Ca^2+^ content than the alkali-activated concretes or mortars; when the OPC matrix comes in contact with CO_2_, CaCO_3_ crystals form that precipitate in the pores, producing a barrier that prevents the diffusion of CO_2._ Therefore, carbonation reactions progress slower in OPC samples, and the carbonation of alkali-activated systems is higher [43,44,45]. In general, given that the matrix of alkali-activated concretes has low or no Ca(OH)_2_ content, the main carbonation reactant has to come from another source. Peter et al. [46], claimed that other constituents in the concrete can participate in the carbonation process, in particular the C–A–S–H/C–S–H gels.

#### 2.2.2. Compressive Strength

**Figure 8:**
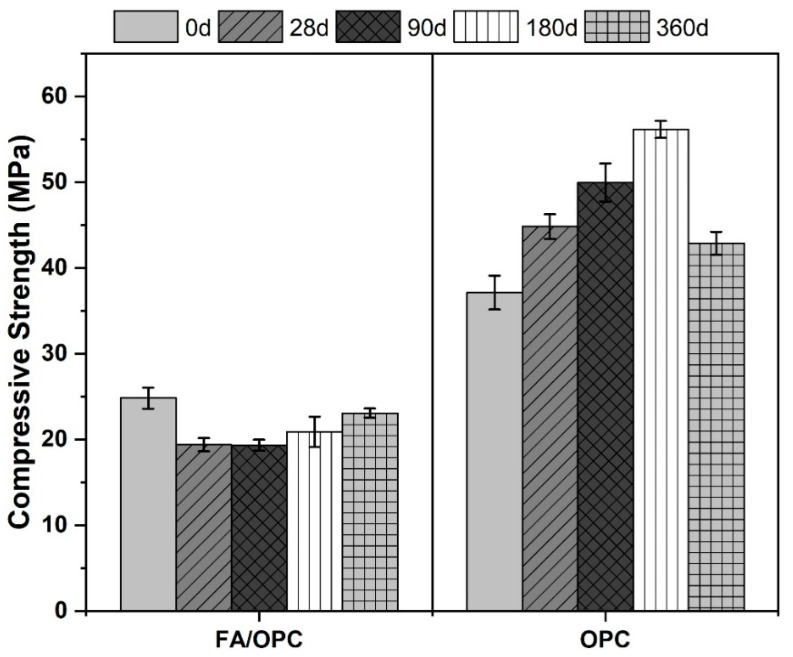
Compressive strength of the concrete at 28, 90, 180 and 360 days of exposure to carbonation.

Figure 8 presents the compressive strength results of the alkali-activated concretes and those made with Portland cement, cured for a period of 28 days and then exposed to carbonation (CO_2_) for 28, 90, 180 and 360 days of exposure. The noncarbonated FA/OPC and OPC concretes exhibited compressive strengths of 24.8 MPa and 37.4 MPa, respectively. During the first 28 days of exposure, the compressive strength of the hybrid alkali-activated concretes decreased by 22% with respect to the noncarbonated concretes, whereas the OPC concretes increased in strength by 20%. Similar tendencies were shown by the specimens exposed for longer periods of time (90 days); for the FA/OPC samples, the loss in strength significantly increased with time. At 180 days of exposure, the FA/OPC concretes had lost the most strength, with a residual strength of 21 MPa. Even though the alkali-activated concretes were completely carbonated, the residual strength is considered appropriate in terms of mechanical behavior for construction material in several civil engineering applications. In addition, a correlation was observed between the carbonation depth and the residual strength that was approximately linear for the concretes; the alkali-activated concretes presented the deepest carbonation depths, as well as the greatest losses in strength. Between 180 and 360 days of exposure, the compressive strengths of the FA/OPC concretes increased, reaching 23 MPa, while a significant loss was observed in the compressive strength of the OPC concretes compared with the samples exposed for 180 days. These results coincide with those reported by Puertas et al. [20], who determined that alkali-activated mortars based on GBFS do not decrease in compressive strength after 4 months of exposure to CO_2,_ and that the OPC mortars do not gain strength after this period.

### 2.3. Exposure to Chlorides

#### 2.3.1. Chloride Permeability

**Figure 9:**
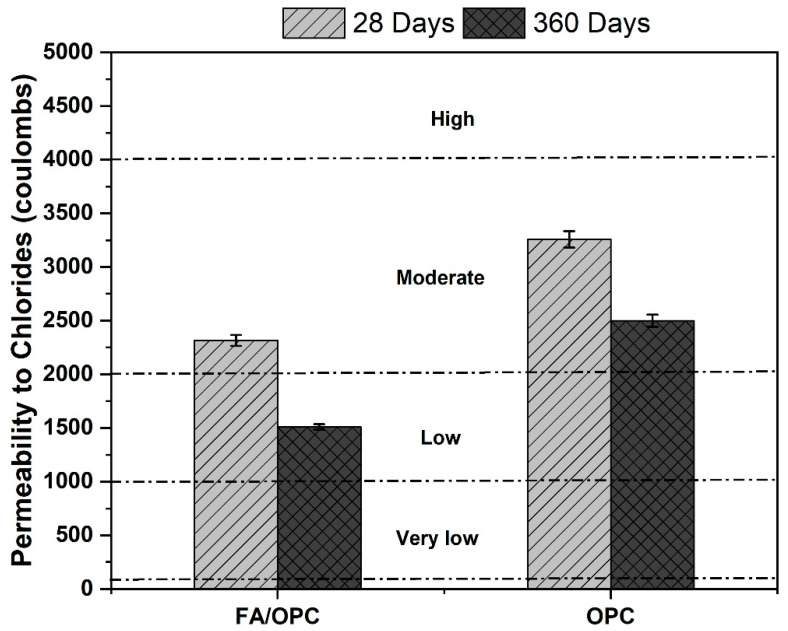
Rapid chloride permeability of the FA/OPC and OPC concrete at 28 and 360 days of curing.

Figure 9 presents the total transferred loads expressed in Coulombs for the concretes under study. In general, a decrease in the chloride permeability was observed with increasing time for all the mixtures.

The OPC concretes had a lower resistance to chloride ion permeability than the hybrid alkali-activated concretes. At the age of 28 days, all the samples were in the moderate permeability range, according to the values specified in the ASTM C1202 standard, by exhibiting the transferred loads of 2315 C and 3256 C for the FA/OPC and OPC concretes, respectively. However, at the curing age of 360 days, the FA/OPC concretes were in the low permeability range, and the OPC concrete remained in the moderate permeability range. In the FA/OPC concretes, reductions in the permeability of 29% and 40% were observed with respect to the standard sample (OPC) for the curing ages of 28 and 360 days, respectively.

In the interpretation of the data obtained by the chloride permeability test (ASTM C1202 standard), we must consider that this test is mainly a measure of the electrical conductivity of the concrete. The ion transport depends to a great extent on the structure of the pore network in the cementing matrix, whereas the electrical conductivity in the concrete is affected by both the structure of the pore network and the chemical composition of the pore solutions [47,48,49]. In the alkali-activated concretes, the pore solutions contain high concentrations of ionic species, mainly Na^+^ and OH^−^ [50,51,52]. The chloride permeability technique indicates the motion of all the ions in the structure, not just the chloride ions. For this reason, the results of the ASTM C1202 tests for alkali-activated concretes could be affected. Any Na^+^ ions present in the pores of the material are expected to diffuse in the direction opposite to that in which the Cl^−^ ions are electrically driven through the pore network, which can lead to a greater load transfer during the test. Therefore, a higher ionic force in the pore solution of the samples is expected to lead to higher values of transferred load, which means that the values observed in the alkali-activated concretes could be higher than the actual chloride permeability.

Table 1 shows the instantaneous resistivity values exhibited by the different concretes analyzed and calculated based on the initial current measured in the chloride permeability test (ASTM C1202). The resistivity increased with curing time, which agrees with the chloride permeability results. Furthermore, the FA/OPC samples presented the best permeability behavior, reaching resistivity values that were 100% and 50% higher than those exhibited by the OPC concretes at ages of 28 and 360 curing days, respectively.

#### 2.3.2. Analysis of XRD and SEM after Chloride Exposition

**Figure 10:**
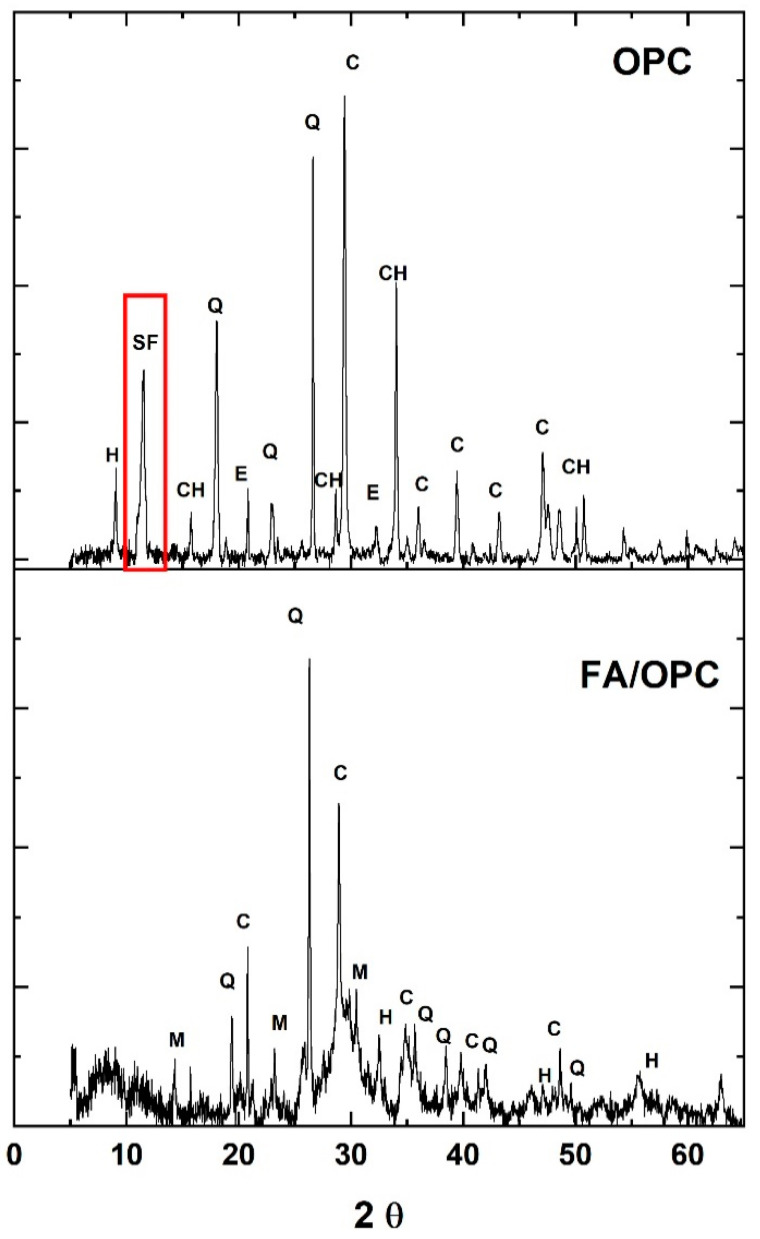
X-ray diffractogram of the FA/OPC and OPC pastes exposed to 3.5% NaCl for a period of 180 days.

Figure 10 presents the X-ray spectra of the FA/OPC and OPC pastes after exposure to NaCl at 3.5% for a period of 180 days. For the OPC samples, hydration products were observed, CH: portlandite, and E: ettringite, as well as the crystalline phases present in the original material, Q: quartz, C: calcite, and H: hydrocalcite. The presence of a new crystalline phase, SF: Friedel’s salt, was observed, which indicates that a chemical bond is the interaction mechanism between the cement and chloride. In the FA/OPC spectrum, crystalline phases corresponding to the raw materials were observed, such as C: calcite, Q: quartz, M: mullite, and H: hematite; the reaction between the chloride and the hybrid FA/OPC alkali-activated system did not change the mineralogical phases observed in Figure 4 (control samples).

**Figure 11:**
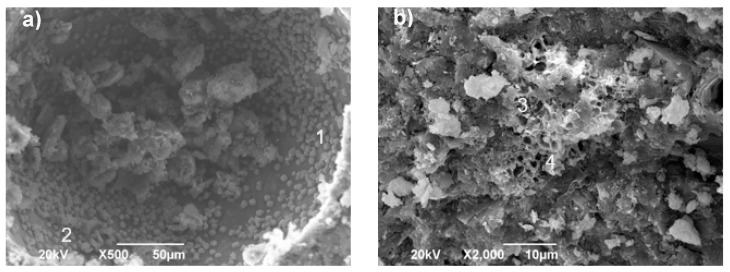
SEM micrographs of the pastes exposed to NaCl for a period of 180 days: (**a**) FA/OPC; and (**b**) OPC.

Figure 11 presents the SEM micrographs of the FA/OPC and OPC pastes exposed for 180 days to NaCl solutions at 3.5%. In the FA/OPC sample, NaCl crystals (halite) were observed embedded in the matrix pores, as shown in Figure 11a. The EDS analysis corroborated the presence of NaCl, as observed in the data reported in Table 2 for points 1 and 2. In the samples corresponding to the OPC pastes, the SEM micrographs and the EDS analyses of points 3 and 4 confirmed the presence of Friedel’s salt, as observed in the X-ray diffraction analysis, as shown in Figure 11b.

## 3. Comparisons of the Results Obtained

Table 3 presents the results obtained in the different tests: the exposure to sulphates, CO_2_ susceptibility and chloride permeability. The concrete performance was classified as excellent, good or bad according to the value obtained in each test.

In general, greater deterioration was observed in the specimens exposed to magnesium sulphate, with expansion percentages of 1.221 and 0.765% for the FA/OPC and OPC concretes, respectively. In contrast, in the presence of sodium sulphate, the expansion percentages were 0.140% and 2.394%, respectively, at 1100 days of exposure, and no loss in strength was observed in the FA/OPC, compared with the 56% loss in strength for the OPC concretes, which was calculated comparing the results with those obtained with the control samples at the same curing age (1100 d, without sulphates). The method used to assess the sulphate attack corresponded to that specified for the mortars in ASTM standard C1012, both for pure and blended Portland cements. This test technique can be considered to be of accelerated character, given that this technique involves exposing specimens by immersion to a solution of Na_2_SO_4_ or MgSO_4_ at a concentration of 50 g/L, which is equivalent to 32,900 and 39,877 ppm sulphate (SO_4_^2^^−^), respectively, values that correspond to very severe exposure (higher than 10,000 ppm) according to building codes. The permeability of chlorides of FA/OPC concrete was 41% of the corresponding to OPC concrete. On the contrary, the hybrid concrete was more susceptible to carbonation than the control sample based on OPC.

## 4. Materials and Methods

### 4.1. Materials

A Colombian fly ash (FA) from a boiler located in a paper mill was used. The chemical composition of these materials presented in Table 4 was determined by X-ray fluorescence (XRF), using a Phillips MagiX-Pro PW-2440 spectrometer equipped with a rhodium tube and a maximum power of 4 KW. It was appreciated that approximately 56.5% of FA is composed of silica, aluminum and iron oxides in addition to a low calcium oxide content (6.68%). The amount of unburnt material was remarkably high (Loss on ignition = 20.67%) and exceeded the standard specification defined in ASTM C618 (6% maximum). It was also relatively high in sodium oxides (7.94%). The crystalline phases identified by X-ray diffraction in the FA were quartz (Q), mullite (M), hematite (H), anhydrite (A), and analcime (An); the apparent density was 2350 kg/m^3^. The particle size analysis was carried out using laser granulometry, obtaining an average size of 22.1 µm and its specific surface area, determined by Brunauer-Emmett-Teller method (BET), was 19.11 m^2^/g. The Portland cement used in the mixtures was type UG (general use); the high LOI content present in OPC was due to the addition of limestone in the production process (estimated percentage 20%). The crystalline phases identified in the OPC were tricalcium silicate (C_3_S), dicalcium silicate (C_2_S), tricalcium aluminate (C_3_A), gypsum (Y) and calcite (C). The apparent density was 3.1 g/cm^3^, and the average particle size was 21.5 µm.

A mixture of commercial sodium silicate (Na_2_SiO_3_.nH_2_O) and industrial sodium hydroxide with 96.7% purity (NaOH) was used as an alkaline activator. The composition of sodium silicate (SS) was SiO_2_: 32.24%, Na_2_O: 11.18% and H_2_O: 55.85%.

### 4.2. Preparation of Specimens and Test Carried Out

The alkali-activated pastes and concretes were made with FA/OPC in a proportion of 80% and 20%, respectively (Table 5). Previously, the synthesis of the binder was realized, and a response surface modelling methodology was used. The factors studied were the SiO_2_/Al_2_O_3_ and Na_2_O/SiO_2_ molar ratios; the ranges selected were 3.0–4.8 and 0.20–0.45, respectively. The compressive strength was used as the response variable. The information obtained was processed using the software Minitab 17. The optimization of the compressive strength was obtained with the relations Si/Al = 4.50 and Na/Si = 0.40. Then, the dosage of the activator in the concrete mixture was adjusted to obtain these molar proportions, taking into account the chemical composition of the precursor and activator. The solution modulus (SiO_2_/Na_2_O) of the activator, which includes the proportion of NaOH and sodium silicate, was 1.10. For the production of the concrete, a coarse aggregate of type siliceous (maximum size: 19 mm, apparent density: 2440 kg/m^3^, and absorption: 2.55%) and a river sand as the fine aggregate (finer module: 3.1, apparent density: 2510 kg/m^3^, and absorption: 1.72%) were used. The proportion of coarse and fine aggregates was 42% and 58%, respectively (see Table 5). The concretes were prepared with a liquid/solid ratio (L/S) of 0.48. It should be clarified that L represents the water content present in the mixture in addition to that provided by the activator, and S includes the solid phase represented by the precursor (FA), cement (OPC) and activator anhydrous. An OPC-based concrete was used as a reference material, as shown in Table 5. FA/OPC samples were cured at room temperature with a relative humidity greater than 90%.

The chemical resistance to sulphate attack was performed on the basis of ASTM C1012/C1012M-18b, exposing the samples to sodium sulphate (Na_2_SO_4_) and magnesium sulphate (MgSO_4_) solutions (5%) at a temperature of 25 °C for up to a period of 1100 days; the pH range of the solutions before use was from 6.0 to 8.0. According to the methodology specified in ASTM C1012, the solution was discarded after the reading of the bars and the mechanical test of the cubes in the standard intervals. Prismatic specimens (50.8 × 50.8 × 285 mm) were used to evaluate the longitudinal expansion and concrete cubes (50.8 × 50.8 × 50.8 mm) to determine the loss of compressive strength (ASTM C109). This loss was calculated by comparing the resistance obtained by the material immersed in the sulphate solution with that obtained at normal curing conditions and at the same curing age. Additionally, a visual inspection of the samples was carried out.

**Figure 12:**
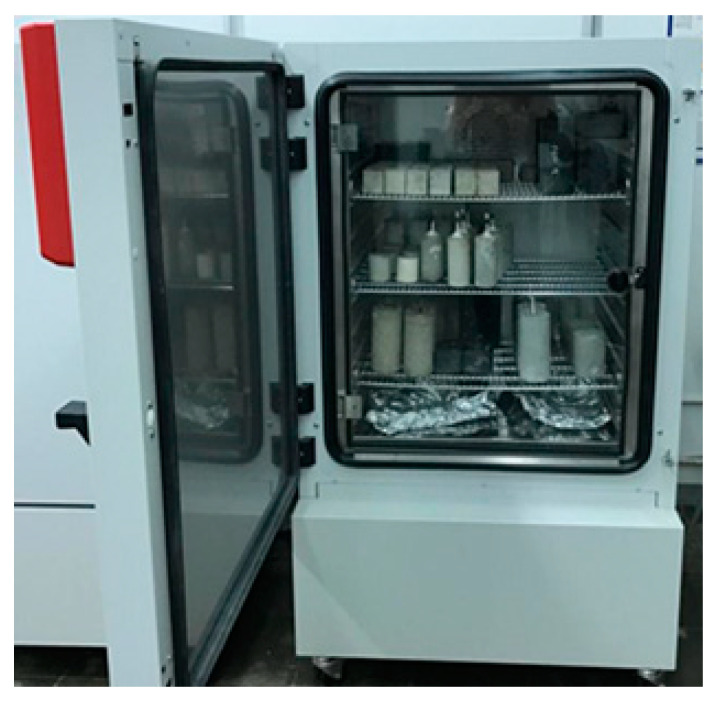
Climate chamber for the CO_2_ exposure under the controlled conditions.

It is known that the carbonation process is a long-term reaction, therefore, an accelerated carbonation system was used to carbonate the concretes (HR = 65%, T = 25 °C and CO_2_ = 1%); for this purpose, a specially designed chamber (Binder KBF 240 UL, 10–70 °C, 10–80% H.R., 1–10% CO_2_) was used as shown in Figure 12. To evaluate the front of carbonation after exposure to CO_2_ concrete, cylinders of 76.2 mm in diameter by 152.4 mm in height were prepared. In order to maintain the radial direction at the ingress of CO_2_, the top and bottom of each of the cylinders was coated with an acrylic resin. Additionally, the loss of compressive strength was evaluated.

The rapid chloride permeability test based on ASTM C1202 was employed to evaluate the performance in chlorides. The initial resistivity of the concretes was determined using the intensity of current at the beginning of the test. In general, the reported values of the tests correspond to the average of three specimens and the trial was conducted at curing ages of 28 and 360 days.

The microstructural analysis was performed on the alkali-activated (FA/OPC) and control pastes (OPC) exposed to the same aggressive conditions—sulfates and chlorides—using X-ray diffraction (XRD) and scanning electron microscopy (SEM) techniques. These tests were realized after 180 days of exposition. The XRD tests were conducted on a PanAnalytical X-ray diffractometer with Cu-radiation in a range of 5–60 degree. The SEM was realized using a JSM 6490LV JOEL with an acceleration voltage of 20 KV, the samples were evaluated in low vacuum mode and an Oxford Instruments Link-Isis X-ray spectrometer was used. The sample for the SEM testing was taken from the center of the fractured sample and the XRD analysis was performed using the rest of the sample. Traducción al Inglés. The XRD samples were previously dried at 50 °C for 24 h and then ground before the test.

## 5. Conclusions

The results obtained in this study confirm that the hybrid alkali-activated concretes (FA/OPC 80/20) evaluated are, in general, less susceptible to sulphate attack than traditional Portland cement concrete at ages of up to 1100 days of exposure. Based on the results, these concretes can be classified as having high resistance to sulphates, specifically to sodium sulphate. In addition, the FA/OPC concretes reported a lesser chloride ion permeability compared with the OPC concretes.

Conversely, the FA/OPC samples were more susceptible to carbonation. However, even though the carbonation coefficients were higher and higher strength losses were observed with respect to the OPC concretes, the residual strength of these samples after 360 days of exposure was 23 MPa with a tendency to stabilize.

The mechanical and durability properties of the hybrid alkali-activated concrete (FA/OPC 80/20), even based on low-quality fly ash, make its use in the civil construction sector more feasible, especially in the prefabrication of blocks and other structural and non-structural elements.

## Figures and Tables

**Table 1:** Resistivity of the FA/OPC and OPC concretes at 28 and 360 days of curing.

Curing Age (Days)	Resistivity (Ω·m)	
	FA/OPC	OPC
28	168.30	84.45
360	198.22	133.65

**Table 2:** EDS spectrum of the samples exposed to NaCl at 3.5.

Points	C	O	Al	Na	Si	Mg	Fe	Ca	Cl	Total
1	-	60.89	6.88	7.06	18.74	-	2.54	2.90	0.99	100
2	-	58.80	7.29	9.36	20.53	-	-	2.11	1.90	100
3	15.95	48.53	4.20	-	3.83	2.00	2.79	19.44	3.25	100
4	-	52.62	3.51	-	4.20	5.19	6.58	21.38	2.50	100

**Table 3:** Concretes performance.

Concretes		FA/OPC	OPC
Compressive strength (MPa)—28 d		24.80	37.40
	Performance test		
**Exposure to sulphates**	Expansion (%) Na_2_SO_4_—1100 d	0.140 (++)	2.394 (−)
	Compressive strength Na_2_SO_4_—1100 d	49.42 (++)	24.19 (−)
	Expansion (%) MgSO_4_—1100 d	1.221 (−)	0.765 (−)
	Compressive strength MgSO_4_—1100 d	21.79 (+)	22.71 (+)
**Exposure to CO_2_**	Carbonation front (mm)—150 d	33.24 (−)	16.4 (+)
	Compressive strength—360 d	23.09 (+)	42.89 (++)
**Exposure to chlorides**	Chloride permeability (coulombs)—360 d	1509 (+)	2499 (−)

(++) Excellent Performance; (+) Good Performance; (−) Bad performance.

**Table 4:** Chemical composition of the materials used: fly ash (FA) and ordinary Portland cement (OPC).

Material	SiO_2_	Al_2_O_3_	Fe_2_O_3_	CaO	MgO	Na_2_O	SO_3_	TiO_2_	LOI *
FA	28.53	19.18	8.80	6.68	2.24	7.94	2.71	1.62	20.67
OPC	19.13	4.42	4.32	57.70	1.60	-	2.32	-	9.78

LOI *: loss on ignition.

**Table 5:** Proportions of the components and liquid/solid ratio (L/S).

Mixes	Kg/m^3^ Concrete						L/S Ratio
	OPC	FA	NaOH	Sodium Silicate	Sand	Gravel	
Reference (OPC)	400	-	-	-	972.7	704.4	0.48
FA/OPC	80	320	48.37	219.66	972.7	704.4	0.48
